# Complete Genome Sequences of H3N2 Canine Influenza Virus with the Matrix Gene from the Pandemic A/H1N1 Virus

**DOI:** 10.1128/genomeA.01010-14

**Published:** 2014-10-02

**Authors:** Minki Hong, Woonsung Na, Minju Yeom, Nanuri Park, Hyoungjoon Moon, Bo-Kyu Kang, Jeong-Ki Kim, Daesub Song

**Affiliations:** a Viral Infectious Disease Research Center, Korea Research Institute of Bioscience and Biotechnology, Daejeon, South Korea; b University of Science and Technology, Daejeon, South Korea; c Green Cross Veterinary Products, Yong-In, South Korea; d College of Pharmacy, Korea University, Sejong, South Korea

## Abstract

We analyzed the complete genome sequence containing the 3′ and 5′ noncoding regions (NCRs) of H3N2 canine influenza virus (CIV) with the matrix gene from the pandemic A/H1N1 virus, which will provide a better understanding of the pathogenesis, transmission, and evolution of variant CIV.

## GENOME ANNOUNCEMENT

Canine influenza virus (CIV), which is a member of the genus *Orthomixoviridae*, family *Orthomixovirideae*, contains single-stranded, negative-sense RNA of the following 8 gene segments: polymerase basic 2 (PB2), polymerase basic 1 (PB1), polymerase acidic (PA), hemagglutinin (HA), nucleoprotein (NP), neuraminidase (NA), matrix (M), and nonstructural (NS). To date, two subtypes of CIV, H3N8, and H3N2 have been isolated from dogs (1, 2). After an outbreak of pandemic influenza A/H1N1 (pH1N1) virus, pH1N1 was transmitted from humans to several species, and many reassortant viruses have been generated (3–6). Recently, A/canine/Korea/MV1/2012 (CIV/H3N2mv) was isolated from a dog, and this reassortant H3N2 CIV carries the matrix gene of the pH1N1 virus. CIV/H3N2mv presented higher viral replication in cells and more efficient direct transmission was observed in ferrets compared to classic CIV H3N2 (7). Therefore, it is necessary to analyze the complete genome sequence containing 3′ and 5′ noncoding regions (NCRs) of CIV/H3N2mv and understand its molecular characteristics.

Viral RNA was extracted from the allantoic fluid of virus-inoculated embryonated chicken eggs with the RNeasy MiniKit (QIAGEN, Inc., Valencia, CA), and the viral RNAs are circularized with T4 RNA ligase as described previously (8, 9). One-step reverse transcriptase-polymerase chain reaction was employed to amplify each of the circularized viral gene segments with the OneStep reverse transcription-PCR (RT-PCR) kit (QIAGEN, Inc.) and gene specific primers. The amplified gene segments were purified and sequenced to determine its exact complete genome sequence containing 3′ and 5′ NCRs by utilizing universal primers (10) with slight modifications and newly designed segment-specific primers.

The complete genome of A/canine/Korea/MV1/2012 (H3N2) is 13,628 nucleotides long. The size of segments 1 (seg-1) to 8 (seg-8) are 2,341, 2,341, 2,233, 1,765, 1,565, 1,467, 1,027, and 889 nt, respectively. They encode 12 viral proteins with the following amino acid lengths: PB2, 759; PB1, 757; PB1-F2, 90; PA, 716; HA, 566; NP, 498; NA, 469; M1, 252; M2, 97; NS1, 230; and NS2 (nuclear export protein [NEP]), 121. The lengths of NCRs of the viral RNA of A/canine/Korea/MV1/2012 (H3N2) were variable (19 [seg-6] to 45 [seg-5] and 20 [seg-7] to 58 [seg-3] nt at the 3′ and 5′ NCRs, respectively) in the different genome segments, but the virus has conserved 12 nucleotides (AGCA/GAAAGCAGG) at 3′ NCR of 8 segments, and 13 nucleotide (CCTTGTTTCTACT) at 5′ NCR, which is consistent with previous studies (11, 12). Here, we described NCR sequences of A/canine/Korea/MV1/2012 (H3N2), and this is the first report of the full-genome sequence containing 3′ and 5′ NCRs of H3N2 CIV carrying the matrix gene of the pH1N1 virus. We hope this information will facilitate further investigation of the pathogenicity, transmission, and evolution of variant CIV.

### Nucleotide sequence accession numbers.

The complete genome sequence of the A/canine/Korea/MV1/2012(H3N2) has been deposited and updated in GenBank under accession numbers KF155142 to KF155149.
